# Supplementary material for: Parapoxvirus Interleukin-10 Homologues Vary in Their Receptor Binding, Anti-Inflammatory, and Stimulatory Activities
Source: Pathogens. 2022 Apr 24;11(5):507. doi: 10.3390/pathogens11050507 (PMC9143231; doi:10.3390/pathogens11050507)
Supplement: Supplementary file 1 [file pathogens-11-00507-s001.zip › pathogens-1686019-supplementary.pdf]

pathogens

MDPI

Supplementary Material

Parapoxvirus Interleukin-10 Homologues Vary in Their Receptor Binding, Anti-Inflammatory, and Stimulatory Activities

Amreen Naqash [1], Gabriella Stuart [1], Roslyn Kemp [2], and Lyn Wise [1,*]

1 Department of Pharmacology and Toxicology, School of Biomedical Sciences, University of Otago, Dunedin, New Zealand.
2 Department of Microbiology and Immunology, School of Biomedical Sciences, University of Otago, Dunedin, New Zealand.
* Correspondence: lyn.wise@otago.ac.nz; Tel.: +64 3 479 7723

Table S1. Human and parapoxvirus IL-10 gene source, loci, length, and cloning details, with protein length and MW estimates after removal of signal peptides

IL-10 source (Virus strain)	GenBank accession no.	Gene locus (CDS)	Gene length (NT)	5′ and 3′ cloning modifications	Protein length (AA)	Protein MW (kDa)
Human	NM_000572.3	(60..596)	537	5′*GGATCC*CACCATGCACAGC′<br>5′*AGATCT*GATAATGAACAGTAG-CAGCAGGAACATCAGCAAA-GCATAGAA3′ [33]	160	18.65
ORFV (NZ2)	DQ184476.1	ORFV127 (127856..128416)	561	5′CGC*TCTAGA*GCCAC-CATGTCGAAGAACAAAATTCT3′<br>5′CGC*TCTAGA*TTACTT-GTCATCGTCGTCCTTGTAG-TCCATTTTAGTAG-TCATGTATG3′ [32]	161	18.87
BPSV (BV-TX09c1)	KM875472.1	BVTX09c1_12 (127282..127842)	561	5′*GGATCC*CACCATGGCTAAT3′<br>5′*AGATCT*GATAGTGAACAGTAG-CAGCAGAACATCAGGCAG-TACATTCAGAG3′	166	19.46
RDPV (HL953)	NC_025963.1	SB87_gp125 (132854..133396)	577	5’*GGATCC*CACCATGGGCAAA3’<br>5′*AGATCT*GATAGTGAACAGTAG CAGCAGGAACATC AGAAAGTAC-CACCA3′	154	17.72
PCPV (F00.120R)	GQ329669.1	ORF001.3 (2186..2785)	607	5’*GGATCC*CACCATGGCTAAC3’<br>5′*ATAGTG*AACAGTAGCAGCAG-GAACATCAGACACAGTACAT3′	177	20.69
GSPV (AFK76s1)	NC_035188.1	CGV03_gp114 (120415..121029)	625	5’*GGATCC*CACCATGGCCTAC3’<br>5′*AGATCT*GATGAACAGTAGCAG-CAGGAACATCAGGAAGTAGAA3′	178	20.51

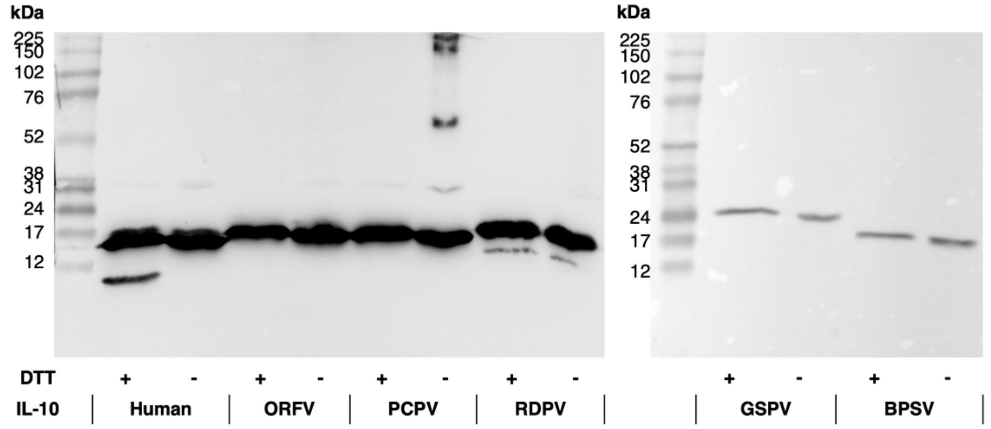

Figure S1. Anti-FLAG Western blot showing protein bands corresponding to the human and parapoxvirus IL-10 proteins. Molecular weight markers and the presence or absence of reducing agent (DTT) are indicated.

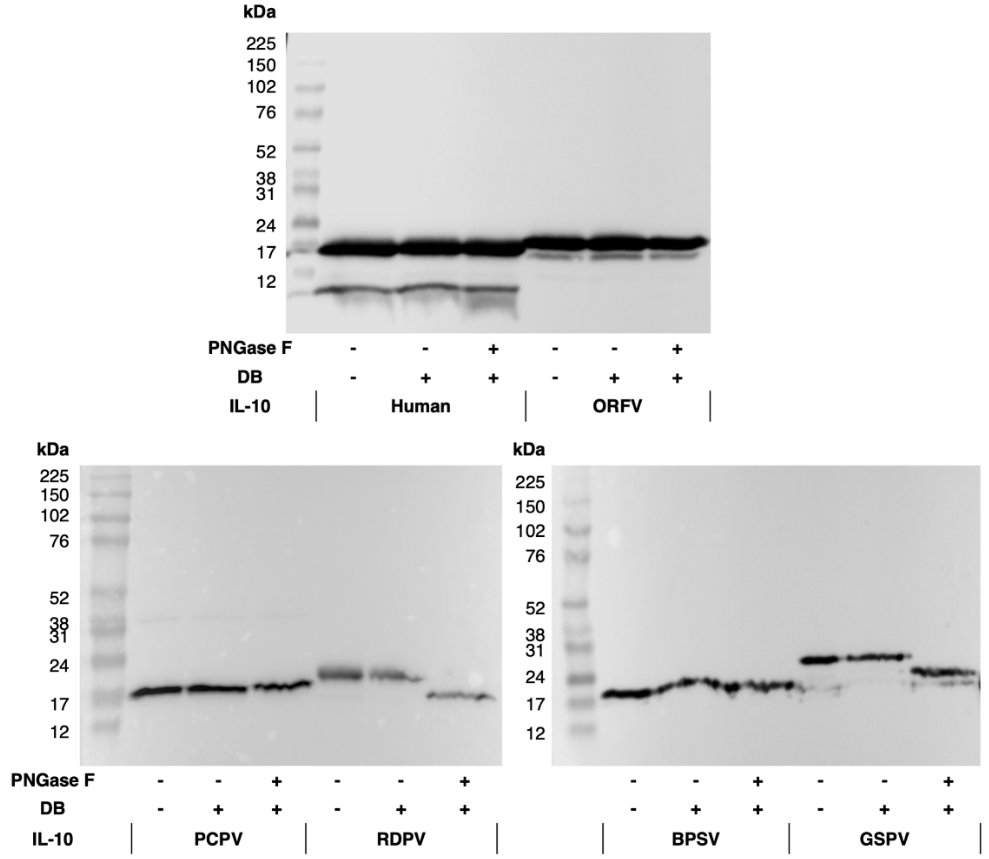

Figure S2. Anti-FLAG Western blot showing bands for human and PPV IL-10 proteins following *N*-glycosidase digestion. Molecular weight markers and the presence and absence of denaturing buffer (DB) and PNGase F are indicated.

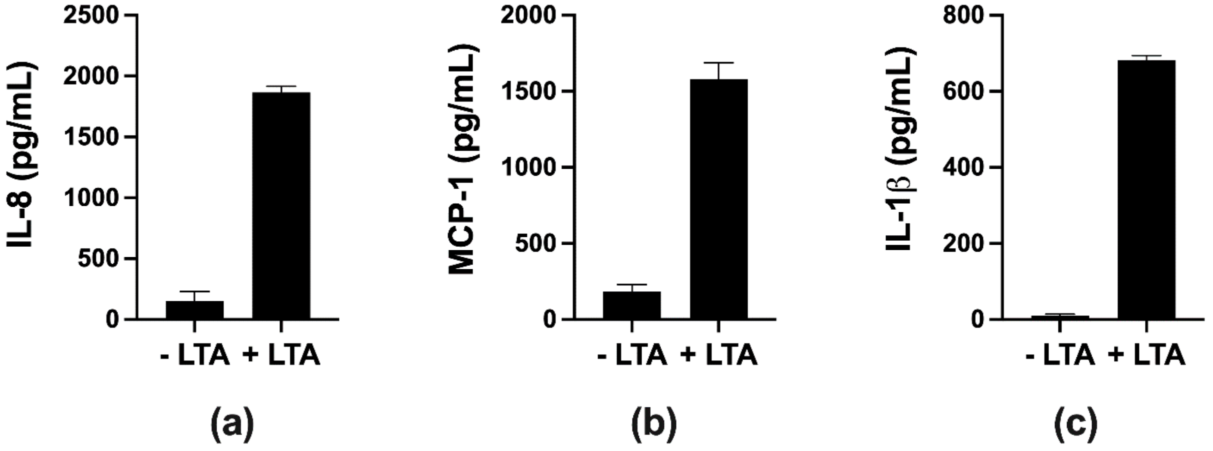

Figure S3. Pro-inflammatory chemokine and cytokine production in stimulated human monocytes. THP-1 cells were stimulated with LTA (+LTA, 1 $\mu g.mL^{-1}$) or left unstimulated (-LTA). After 24, 24, or 48 h incubation, ELISA was used to assess the levels of IL-8 (a), MCP-1 (b), IL-1β (c), respectively. Values represent the mean ± SEM of three independent experiments.

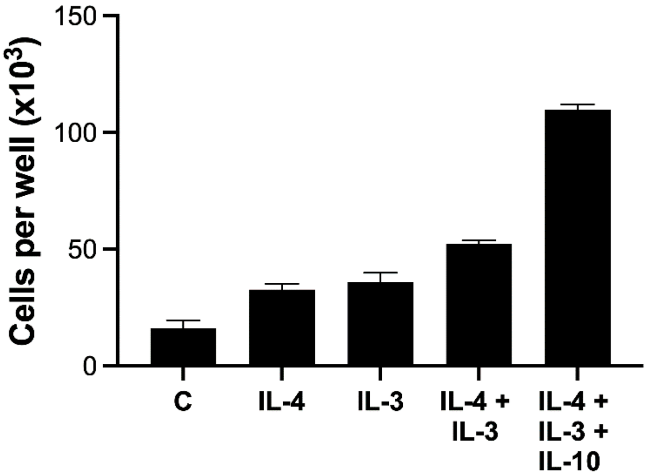

Figure S4. Stimulation of murine mast cell proliferation. MC/9 cells were co-stimulated with IL-4-conditioned medium from concanavalin A-activated murine splenocytes (IL-4), IL-3-containing conditioned medium from murine WEHI-3 cells (IL-3) and/or human IL-10 (IL-10). After 48 h incubation, the cell number was assessed through tetrazolium dye reduction and a standard curve. Values representing the mean ± SEM of three independent experiments.
